# A new biomarker in severe pneumonia associated with coronavirus disease 2019: hypoalbuminemia. A prospective study

**DOI:** 10.1590/1516-3180.2021.0066.R2.16082021

**Published:** 2022-03-18

**Authors:** Yavuz Otal, Gamze Avcıoglu, Fadime Gullu Haydar

**Affiliations:** I MD. Physician, Department of Emergency Medicine, Ankara City Hospital, Ankara, Turkey.; II MD. Physician, Department of Medical Biochemistry, Karadeniz Ereğli State Hospital, Zonguldak, Turkey.; III MD. Physician, Department of General Surgery, Ankara City Hospital, Ankara, Turkey.

**Keywords:** Biomarkers, Pneumonia, Coronavirus, Hypoalbuminemia, Emergency medicine, General surgeon, Medical biochemistry, Pneumonitis

## Abstract

**BACKGROUND:**

Effective triage and early detection are very important for controlling and treating coronavirus disease 2019 (COVID-19). Thus, the relationships between hypoalbuminemia and other acute-phase reactants in such cases need to be evaluated.

**OBJECTIVES:**

To investigate the importance of albumin levels in cases of severe pneumonia due to COVID-19.

**DESIGN AND SETTING:**

Prospective study conducted in Ankara City Hospital (a stage 3 hospital), Turkey.

**METHODS:**

Data from 122 patients diagnosed with pneumonia due to COVID-19 who were admitted to this hospital were analyzed statistically in comparison with date from 60 healthy controls. Three groups were established: healthy controls, intubated patients and non-intubated patients. Lung tomography scans from the patients were examined one-by-one. Real-time polymerase chain reaction (RT-PCR) test results were recorded.

**RESULTS:**

Albumin levels were statistically significantly lower in the intubated and non-intubated groups than in the control group, in comparing the three groups (P *<* 0.01). The other acute-phase reactants, i.e. neutrophil-to-lymphocyte ratio and C-reactive protein levels, were significantly higher in the intubated and non-intubated groups than in the control group (P < 0.05). Albumin levels were also significantly lower in the intubated group than in the non-intubated group (P = 0.02). No differences were detected with regard to other parameters (P > 0.05).

**CONCLUSIONS:**

Hypoalbuminemia may constitute a biomarker indicating the severity of pneumonia due to COVID-19.

## INTRODUCTION

Coronavirus disease 2019 (COVID-19) was caused by a novel coronavirus infection called severe acute respiratory syndrome coronavirus 2 (SARS-CoV-2).^1^ The infection spread rapidly all over the world and was declared to be a pandemic by the World Health Organization. A total of 78,604,532 documented cases had been reported worldwide as of December 26, 2020, and 1,744,235 patients had died.^2^ Since there is no current specific treatment and drug against this novel virus, it is very important to determine the risk factors for severe prognosis.

Lung involvement is a serious complication in severe infection and requires hospitalization in an intensive care unit. Usually, the lungs are bilaterally affected. Treatment with mechanical ventilation may be necessary as a result of severe respiratory failure. There is no special drug for treatment of these patients, and supportive treatments are applied. The blood parameters of these patients can be variable. Examination of blood parameters in cases of severe pneumonia due to COVID-19 is providing a guide to prognosis and treatment in cases with comorbidities.

Albumin is a protein synthesized by the liver plays important roles in maintaining nutrition and plasma osmolality.^3^ Serum albumin, C-reactive protein (CRP), white blood cell (WBC) and neutrophil-to-lymphocyte (N/L) ratio values are known to be acute-phase reactants. The WBC, CRP and N/L ratio are objective systemic inflammation markers that are usually taken to measure the susceptibility of the patient to mortality.^4,5,6^ In our study, the relationships between these acute-phase reactants were compared in cases of severe pneumonia due to COVID-19.

## OBJECTIVES

Serum albumin, C-reactive protein, white blood cell and neutrophil-to-lymphocyte ratio values are known to be acute-phase reactants. Their levels change during acute-period events in metabolism. Changes to in the levels of these biomarkers during the acute period of COVID-19 pneumonia can provide us with information about the severity of the disease and can be used to predict the prognosis.

In our study, the relationships between these acute-phase reactants were compared in cases of severe pneumonia due to COVID-19. Effective triage and early detection are very important for controlling and treating this disease. For this purpose, the relationship between hypoalbuminemia and other acute-phase reactants was compared in cases of severe pneumonia due to COVID-19.

## METHODS

Approval was obtained from the Ethics Committee of Ankara City Hospital (date: April 14, 2021; number: 14.04.2021/1527). The blood samples were taken from 122 patients and 60 healthy volunteers and were evaluated using a computer after working in the laboratory.

The subjects were divided into three groups: group 1: healthy controls; group 2: intubated; and group 3: non-intubated. Lung computed tomography (CT) scans from the patients who were diagnosed with COVID-19 were examined one-by-one. Patients who were diagnosed with COVID-19 as a result of lung tomography reports were included in the study. Polymerase chain reaction (PCR) results from the analysis system were reviewed. The tomography findings from 42 PCR-negative patients were compatible with presence of COVID-19. These patients were included in the study.

Albumin, CRP, WBC and N/L ratio values of all the cases included in the study were separately entered into the statistics software. Individuals who were under the age of 18 years, trauma patients, and pregnant women were excluded from the study.

### Statistical analysis

Statistical analyses were done by using the IBM SPSS Statistics (version 22) computer software (IBM, Armonk, United States, 2011). The distribution of the variables was examined by using the Kolmogorov-Smirnov and Shapiro-Wilk tests. The quantitative data were expressed as the mean ± standard deviation (SD) or the median and interquartile range (IQR). One-way analysis of variance (ANOVA), with Tukey’s post-hoc test, and the independent-group Student t test were applied to the data that showed normal distribution. The Kruskal-Wallis, Mann-Whitney U and Dunn post-hoc tests were applied to the data that did not show normal distribution. Chi-square testing was performed on categorical data. P-values < 0.05 were considered statistically significant.

## RESULTS

**Figure 1.**
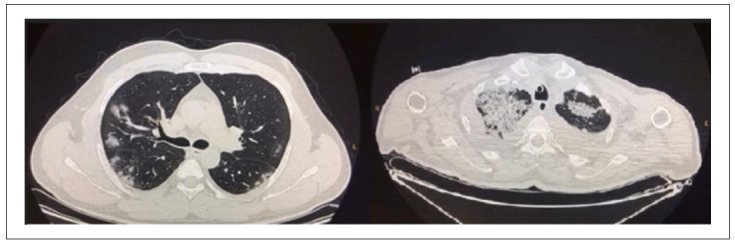
Computed tomography (CT) sample images of patients with COVID-19.

The demographic data of the pneumonia cases due to COVID-19 and the control group are presented in Table 1. No significant differences were detected with regard to age or gender. Among the 122 patients, 80 were not intubated and 42 were intubated and treated with mechanical ventilation. PCR test results were negative in the cases of 42 patients and positive for 80 patients. The tomography scans of all the patients were examined and the findings were entered into the system. The tomography findings from 42 PCR-negative patients were compatible with COVID-19. The tomography results from the two different patient groups with COVID-19 pneumonia are shown in Figure 1.

**Table 1.** Characteristics of routine blood parameters of the three groups: healthy controls, intubated patients and non-intubated patients

Characteristics	Control group (n = 60)	Non-intubated group (n = 80)	Intubated group (n = 42)	P-value*	P-value**
**Age in years: median (IQR), range**	40.0 (29-72), 18-79	70.0 (57-75), 23-100	80.0 (73-87), 55-100	0.361	0.693
**Gender: male/female**	34/26	49/31	25/17	0.861	0.876
**Laboratory analysis: median (IQR)**					
Na, mEq/l	140 (138-142)	138 (135-141)	138 (134-141)	0.236	0.495
K, mEq/l	4.2 (4.0-4.5)	4.2 (3.9-4.7)	4.3 (3.8-4.5)	0.194	0.221
Ca, mg/dl	9.1 (9.0-9.4)	8.6 (8.1-9.3)	8.5 (7.8-9.0)	0.110	0.820
Glucose, mg/dl	98 (85.25-100.2)	115 (95.8-168.5)	118.5 (96.5-165)	0.003	0.222
LDH, U/l	198 (172-244.5)	308 (217.5-454.0)	323.5 (204-460)	0.006	0.951
WBC ×10^9^/l	7.75 (6.7-1.7)	7.3 (5.4-10.8)	7.1 (5.5-9.6)	0.341	0.460
Hemoglobin, g/dl	13.8 (12.7-14.5)	12.0 (10.6-13.6)	11.9 (9.9-12.9)	< 0.001	0.795
PLT ×10^9^/l	250 (200.8-304.5)	251.5 (160.5-340)	235.5 (148.5-317.3)	0.584	0.570
N/L ratio	2.1 (1.4-5.4)	5.9 (3.4-12.8)	6.9 (2.9-11.7)	0.002	0.411
Albumin, g/dl	36.0 (44.0-48.0)	35.0 (32.0-39.0)	31.0 (27.8-35.0)	< 0.001	0.002
CRP, g/l	0.03 (0.02-0.07)	0.05 (0.01-0.12)	0.08 (0.03-0.12)	0.041	0.807
**Comorbidities**					
Systemic hypertension, n (%)	–	24 (19.7%)		–	–
Diabetes mellitus, n (%)	–	25 (20.5%)		–	–
Ischemic heart disease, n (%)	–	34 (27.9%)		–	–
Chronic renal disease, n (%)	–	5 (4.1%)		–	–
Cancer, n (%)	–	4 (3.3%)		–	–
Cerebrovascular events, n (%)	–	4 (3.3%)		–	–
Chronic obstructive pulmonary disease, n (%)	–	5 (4.1%)		–	–
Neurological disease (Alzheimer, Parkinson etc.)	–	9 (7.4%)		–	–
Other, n (%)	–	12 (9.8%)		–	–

IQR = interquartile range; Na = sodium; K = potassium; Ca = calcium; LDH = lactate dehydrogenase; WBC = white blood cell; PLT = platelet; N/L ratio = neutrophil-to-lymphocyte ratio; CRP = C-reactive protein. P-values less than 0.05 were considered significant and are highlighted in bold. *Comparison of three groups; **Comparison of intubated and non-intubated groups.

**Figure 2.**
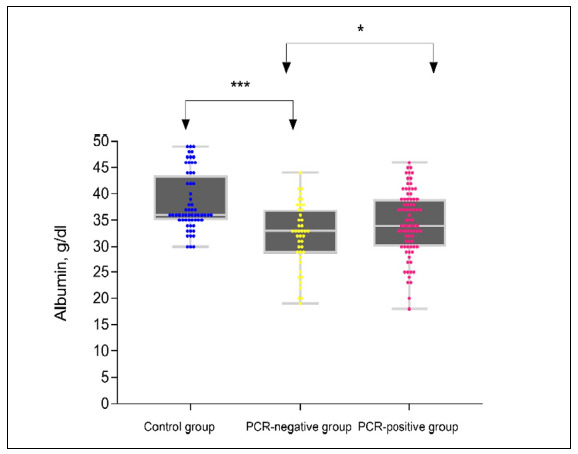
Albumin levels in the three groups (healthy controls, PCR-negative patients and PCR-positive patients) according to polymerase chain reaction (PCR) results.

**Figure 3.**
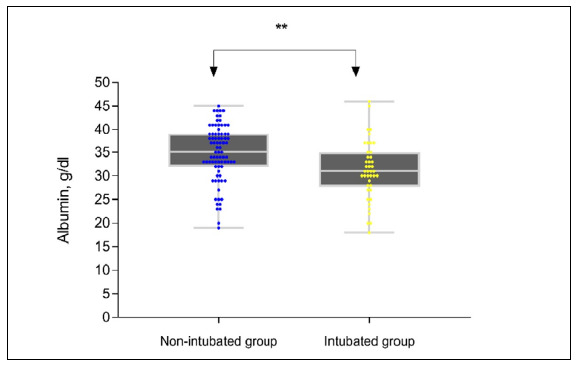
Albumin levels of the patient groups according to intubation.

The P-value was calculated for each parameter by making comparisons between the groups in the statistical analyses. From examination of the data of the three groups, it was found that the albumin levels were lower in the intubated and non-intubated groups than in the control group, at statistically significant levels (P *<* 0.01). The distribution of the data is shown in Figures 2 and 3. The albumin levels were lower in patients with COVID-19 pneumonia who received negative results from the PCR (Figure 2). Also, comparison of the intubated and non-intubated groups showed that the albumin levels were significantly lower in the intubated and non-intubated groups, as seen in Table 1 (P = 0.02). Albumin levels were also found to be significantly lower in the intubated group than in the non-intubated group, as shown in Figure 3 (P = 0.02).

**Figure 4.**
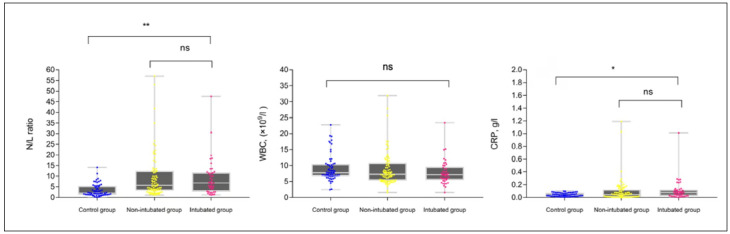
C-reactive protein (CRP), neutrophil-to-lymphocyte (N/L) ratio and white blood cell (WBC) levels of the three groups (healthy controls, non-intubated patients and intubated patients).

When the other acute-phase reactants, i.e. N/L ratio and CRP level were compared among the three groups, they were found to be significantly higher in the intubated and non-intubated groups (P < 0.05). However, no differences were detected between the intubated and non-intubated groups (P > 0.05). The WBC values did not show any statistically significant differences among the three groups or between the two groups (intubated and non-intubated) (Figure 4).

Comparison of the intubated and non-intubated groups without involving the control group showed that there was only a statistically significant difference in the albumin levels (P = 0.002). As demonstrated in Figure 3, the albumin levels were found to be even lower in intubated cases than in non-intubated cases. No significant differences were detected with regard to CRP, WBC and N/L ratio (P > 0.05).

## DISCUSSION

In our study investigating the role of acute-phase reactants in the etiopathogenesis of COVID-19 pneumonia, the low serum albumin level in these cases makes us think that hypoalbuminemia may be a biomarker. The data relating to WBC, CRP and N/L ratio were similar to those reported in the literature. Hypoalbuminemia was seen to be an important prognostic criterion in cases of mortality among individuals with COVID-19 pneumonia. Adding albumin to the treatment may be effective in reducing mortality in cases of hypoalbuminemia, which we think may constitute a biomarker showing the severity of the disease.

The mechanisms of hypoalbuminemia in COVID-19 have not been fully investigated or explained. In some studies, hypoalbuminemia has been detected in severe COVID-19 cases. There is a possibility that the coronavirus may also affect liver functions, and thus may be as the reason for the low serum albumin levels in cases of severe COVID-19 pneumonia. Therefore, hypoalbuminemia may suggest the presence of liver dysfunction.^7^

COVID-19 is a pandemic that first appeared in Wuhan, China, in December 2019, and has spread all over the world. There is still no specific treatment for the disease and vaccine studies are still continuing. Patients with severe bilateral lung involvement are intubated and treated with mechanical ventilation. This is done especially in cases of patients with blood oxygen levels falling below certain levels. Supportive treatments are applied in such cases of severe pneumonia due to COVID-19, which might otherwise lead to death.

Albumin is a protein that is synthesized by the liver and plays important roles in maintaining nutrition and plasma osmolality.^3^ Li et al. argued that low albumin levels are indicative of poor nutritional status and also reduce the immunity of the body. Furthermore, they reported that the immune response of the host to ribonucleic acid (RNA) virus infection is often weakened because of the nutritional insufficiency, which is not always taken into consideration in making the clinical diagnosis and implementing the treatment.^7^ Comorbid conditions may be the cause of low albumin levels in patients diagnosed with COVID-19 and hypoalbuminemia.

Hypoalbuminemia is related to inflammation. Inflammation causes expansion of the interstitial gap and the volume of albumin distributed increases through increased capillary permeability, with escape of serum albumin. It has been shown that the half-life of albumin is shortened and that the total albumin mass is reduced. These two factors cause hypoalbuminemia, despite increased fractional synthesis rates in the plasma. For this reason, hypoalbuminemia stems from inflammatory conditions that prevent adequate responses to events, such as surgery or chemotherapy, and is associated with poor quality of life and decreased survival. Decreased serum albumin levels are indicative of clinical deterioration. Albumin acts as a main extracellular cleaner, antioxidant and supplier of amino acids for cell synthesis. Management of hypoalbuminemia needs to be based more on correcting the causes of the continuing inflammation than on albumin infusion.^8–10^ The inflammation in cases of pneumonia that develop due to COVID-19 can cause hypoalbuminemia. Improvement of states of hypoalbuminemia through COVID-19 treatment can contribute to reducing the inflammation.

In the present study, the albumin levels were low in the analyses made in the two groups diagnosed with COVID-19 pneumonia (intubated and non-intubated), without involving the control group. The lack of significant differences in other parameters in the comparisons between these two groups suggests to us that hypoalbuminemia is more important for showing the severity of the disease. The significantly lower albumin levels in the intubated group than in the non-intubated group suggests to us that these lower levels show the greater severity of the disease. Addition of albumin to the treatment, in cases of detection of hypoalbuminemia in intubated patients, may contribute to the recovery. Wider studies are needed on this issue.

States of comorbidity that can lower serum albumin levels may contribute to hypoalbuminemia in the COVID-19 pandemic. These conditions are encountered in intensive care patients. The presence of comorbidities may adversely affect the treatment process. In some published papers, it was reported that serum albumin levels were negatively affected in situations of hypervolemia.^8–10^ Cases of COVID-19 pneumonia with hypervolemia may be more affected. Avoiding hypervolemia can contribute to recovery. Conditions such as hypervolemia, proteinuria or liver failure, which may decrease serum albumin levels in COVID-19 pneumonia, should be treated.

It was reported in some previous studies that hypoalbuminemia, lymphopenia, decreased lymphocyte and neutrophil percentages, high C-reactive protein levels and high lactate dehydrogenase (LDH) levels were common laboratory abnormalities.^11,12,13,14^ It was also found that serum albumin values, lymphocyte cell counts and percentages, neutrophil percentages and LDH and CRP levels were highly correlated with acute lung damage.^8,9^ These conditions can play critical roles in cases of severe pneumonia that develop due to COVID-19. In our study, it was found that albumin values were low, compared with those of the control group. Also, the CRP levels and N/L ratios were high and the WBC values were not significant. Moreover, our findings were in line with those in the literature.

We believe that an idea of the severity of the disease of this pandemic, which has affected the entire world, can be obtained in terms of lower albumin levels in cases of greater severity of lung involvement. Treatments for hypoalbuminemia can contribute to improving the condition of patients with pneumonia.

### Limitations

Our study population was small. Serum albumin levels are also affected by conditions such as hypervolemia. It needs to be asked whether serum albumin levels might also cause worsening of COVID-19 pneumonia accompanied by hypervolemia, and whether these levels might have a role in etiopathogenesis. More extensive studies may be required.

## CONCLUSION

Hypoalbuminemia may constitute a biomarker indicating the severity of cases of pneumonia due to COVID-19.

## References

[1] Coronaviridae Study Group of the International Committee on Taxonomy of Viruses (2020). The species severe acute respiratory syndrome-related coronavirus: classifying 2019-nCoV and naming it SARS-CoV-2. Nat Microbiol..

[2] World Health Organization homepage https://www.who.int/.

[3] Bernardi M, Angeli P, Claria J (2020). Albumin in decompensated cirrhosis: new concepts and perspectives. Gut..

[4] Kalra L, Smith CJ, Hodsoll (2019). Elevated C-reactive protein increases diagnostic accuracy of algorithm-defined stroke-associated pneumonia in afebrile patients. Int J Stroke..

[5] Li W, Ai X, Ni Y, Ye Z, Liang Z (2019). The Association Between the Neutrophil-to-Lymphocyte Ratio and Mortality in Patients with Acute Respiratory Distress Syndrome: A Retrospective Cohort Study. Shock..

[6] Lu A, Li H, Zheng Y (2017). Prognostic Significance of Neutrophil to Lymphocyte Ratio, Lymphocyte to Monocyte Ratio, and Platelet to Lymphocyte Ratio in Patients with Nasopharyngeal Carcinoma. Biomed Res Int..

[7] Huang J, Cheng A, Kumar R (2020). Hypoalbuminemia predicts the outcome of COVID-19 independent of age and co-morbidity. J Med Virol..

[8] Soeters PB, Wolfe RR, Shenkin A (2019). Hypoalbuminemia: Pathogenesis and Clinical Significance. JPEN J Parenter Enteral Nutr..

[9] Fulks M, Stout RL, Dolan VF (2010). Albumin and all-cause mortality risk in insurance applicants. J Insur Med..

[10] Goldwasser P, Feldman J (1997). Association of serum albumin and mortality risk. J Clin Epidemiol..

[11] Liu Y, Yang Y, Zhang C (2020). Clinical and biochemical indexes from 2019-nCoV infected patients linked to viral loads and lung injury. Sci China Life Sci..

[12] Bannaga AS, Tabuso M, Farrugia A (2020). C-reactive protein and albumin association with mortality of hospitalised SARS-CoV-2 patients: A tertiary hospital experience. Clin Med (Lond)..

[13] Afari ME, Bhat T (2016). Neutrophil to lymphocyte ratio (NLR) and cardiovascular diseases: an update. Expert Rev Cardiovasc Ther..

[14] Wang L (2020). C-reactive protein levels in the early stage of COVID-19. Med Mal Infect..

